# Using torsional wave elastography to evaluate spring pot parameters in skin tumor mimicking phantoms

**DOI:** 10.1038/s41598-024-66621-w

**Published:** 2024-07-11

**Authors:** Yousef Almashakbeh, Hirad Shamimi, Antonio Callejas, Guillermo Rus

**Affiliations:** 1 Department of Allied Engineering Sciences, Facility of Engineering, The Hashemite University, Zarqa, 13133 Jordan; 2 Department of Structural Mechanics, University of Granada, Granada, 18071 Spain; 3 Instituto de Investigación Biosanitaria, ibs.GRANADA, Granada, 18012 Spain; 4 Excellence Research Unit, “Modelling Nature” (MNat), Granada, 18071 Spain

**Keywords:** Biomedical engineering, Skin cancer

## Abstract

Estimating the tissue parameters of skin tumors is crucial for diagnosis and effective therapy in dermatology and related fields. However, identifying the most sensitive biomarkers require an optimal rheological model for simulating skin behavior this remains an ongoing research endeavor. Additionally, the multi-layered structure of the skin introduces further complexity to this task. In order to surmount these challenges, an inverse problem methodology, in conjunction with signal analysis techniques, is being employed. In this study, a fractional rheological model is presented to enhance the precision of skin tissue parameter estimation from the acquired signal from torsional wave elastography technique (TWE) on skin tumor-mimicking phantoms for lab validation and the estimation of the thickness of the cancerous layer. An exhaustive analysis of the spring-pot model (SP) solved by the finite difference time domain (FDTD) is conducted. The results of experiments performed using a TWE probe designed and prototyped in the laboratory were validated against ultrafast imaging carried out by the Verasonics Research System. Twelve tissue-mimicking phantoms, which precisely simulated the characteristics of skin tissue, were prepared for our experimental setting. The experimental data from these bi-layer phantoms were measured using a TWE probe, and the parameters of the skin tissue were estimated using inverse problem-solving. The agreement between the two datasets was evaluated by comparing the experimental data obtained from the TWE technique with simulated data from the SP- FDTD model using Pearson correlation, dynamic time warping (DTW), and time-frequency representation. Our findings show that the SP-FDTD model and TWE are capable of determining the mechanical properties of both layers in a bilayer phantom, using a single signal and an inverse problem approach. The ultrafast imaging and the validation of TWE results further demonstrate the robustness and reliability of our technology for a realistic range of phantoms. This fusion of the SP-FDTD model and TWE, as well as inverse problem-solving methods has the potential to have a considerable impact on diagnoses and treatments in dermatology and related fields.

## Introduction

The epidermis, the outermost layer of the skin, is susceptible to uncontrolled cellular proliferation of abnormal cells, leading to skin cancer. This condition arises from unrepaired DNA damage that precipitates mutations, compelling skin cells to proliferate rapidly and form cancerous tumors^1^. In the United States, the prevalence of skin cancer exceeds that of all other cancers combined, making it the most common malignancy worldwide^2^. Statistically, one in five individuals will develop skin cancer by the age of 70^3^. Early diagnosis and treatment are crucial, as most cases are treatable in their initial stages.

Mechanical analysis of human skin is a significant obstacle in many fields. For example, the quantification of the mechanical properties of human skin is helpful in the diagnosis of skin cancer. Several non-invasive methods have been developed for the assessment of skin mechanical properties. The most frequently utilized methods for measurement include suction^4,5^, torsion^6,7^, and traction^8^. The main disadvantage of these methods is the alteration of the skin’s natural state of tension. This is because the experimental device needs to be attached to the epidermis while being tested. As a result, it is extremely difficult to estimate and extract the preload value induced by mechanical devices. This can affect the measured values of mechanical properties.

Historically, various elastography techniques have been utilized since the late 20th century to assess tissue stiffness, an important parameter in identifying pathological changes within tissues. Quantitative elastography, in particular, measures shear stiffness by evaluating shear wave velocity. Static (SE) and dynamic (DE) elastography have been instrumental in quantifying tissue stiffness^9^, though each method has its limitations. SE’s accuracy is affected by unspecified applied pressure and its inability to probe deep tissue spots, while DE requires a higher energy input, posing risks to small and delicate organs. To overcome these limitations, TWE was developed. TWE focuses on the propagation of shear elastic waves into tissues and is particularly suited for radial application in skin tissues. This technique represents a significant advancement over traditional dynamic elastography methods by allowing for precise evaluation of soft tissue mechanical properties, especially in cylindrical geometries^10^. The principle that tumors exhibit greater stiffness compared to healthy tissue underlies the rationale for using elastography in cancer detection. This increased stiffness results from collagen remodeling in pathological tissues. Research has consistently shown differences in mechanical characteristics between normal, malignant, and benign tissues, with Young’s modulus for cancerous breast tissue being significantly higher than that of normal or benign tissue^11^.

Modeling the viscoelastic properties of tissues has traditionally involved the use of rheological models such as the Maxwell (M), Generalized Maxwell (GM), Zener (Z), and Kelvin-Voigt (KV) models. However, these classical models have often fallen short in accurately describing the complex viscoelastic behavior of biological tissues over various time scales and frequencies^12,13^. In response to these limitations, the SP model has emerged as a significant alternative. This model leverages fractional calculus to more precisely represent the viscoelastic dynamics of tissues, thanks to its inherent power-law response in both time and frequency domains^14^. The use of the SP model in conjunction with the FDTD method for simulating elastic wave propagation offers a novel approach to studying the mechanical properties of skin tissues affected by cancer^9^.

Our research aims to validate SP biomarkers in tissue-mimicking phantoms and to reconstruct the viscoelastic properties of bi-layer phantoms using experimental data and the SP model solved by the FDTD method. This paper is structured to first introduce the problem and the innovative approach taken. It then details the materials and methods, including the development of a skin tissue model, the preparation of tissue-mimicking phantoms, and the comparison of TWE results with the gold-standard techniques. Finally, it discusses the findings and concludes the study, highlighting the potential of this method in advancing the diagnosis and understanding of skin cancer.

## Materials and methods

**Figure 1:**
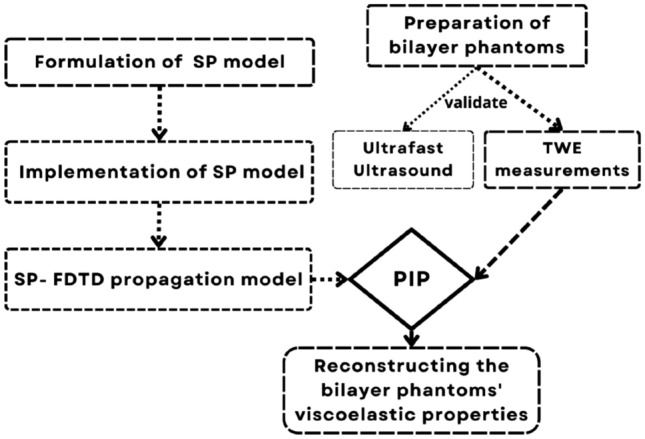
Validation flowchart for the spring-pot viscoelastic model.

The methodology that has been proposed in this research includes four steps (see Fig. 1). The first derivation and implementation of the SP model equations. Putting together the numerical setup and optimization of the proposed model. Third, experimental validation of computational modeling and finally, reconstructing the mechanical properties of skin tissue and TWE using the inverse problem.

### Rheological models

**Figure 2:**
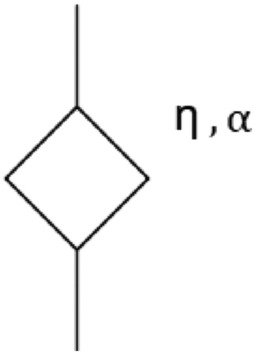
Spring pot model configuration.

As with many soft tissues, the skin tissue can be modeled as a linear viscoelastic medium^15^. The long history of biomechanics, including the broad relaxation spectrum, evidence from viscoelastic soft tissues across a wide range of time and frequency, and the theoretical framework of multiple relaxations all contribute to this conclusion^16^. All models that capture the multi-scale nature of biological tissues lead to fractional derivative models^16^. So, the use of SP model is evaluated in this study (see Fig. 2).

The viscoelastic tissue properties can be retrieved by fitting the data to a mechanical tissue model using the curve as a reference. Complex shear modules for SP^17^ are:

1 $$\begin{aligned} G_{sp} = \eta \omega ^{\alpha } \cos \left( \frac{\alpha \pi }{2}\right) + i \eta \omega ^{\alpha } \sin \left( \frac{\alpha \pi }{2}\right) \end{aligned}$$

where $\eta$ is the shear viscosity (in Pa s). The relationship between shear wave velocity and the complex shear modulus is well established for mass density $\rho$^18^ to plot the Dispersion curves.

2 $$\begin{aligned} C_s(\omega ) = \sqrt{\frac{2\left( \dot{G}^2+\ddot{G}^2\right) }{\rho \left( \dot{G}+\sqrt{\dot{G}^2+\ddot{G}^2}\right) }} \end{aligned}$$

### Formulation of the spring-pot model

**Figure 3:**
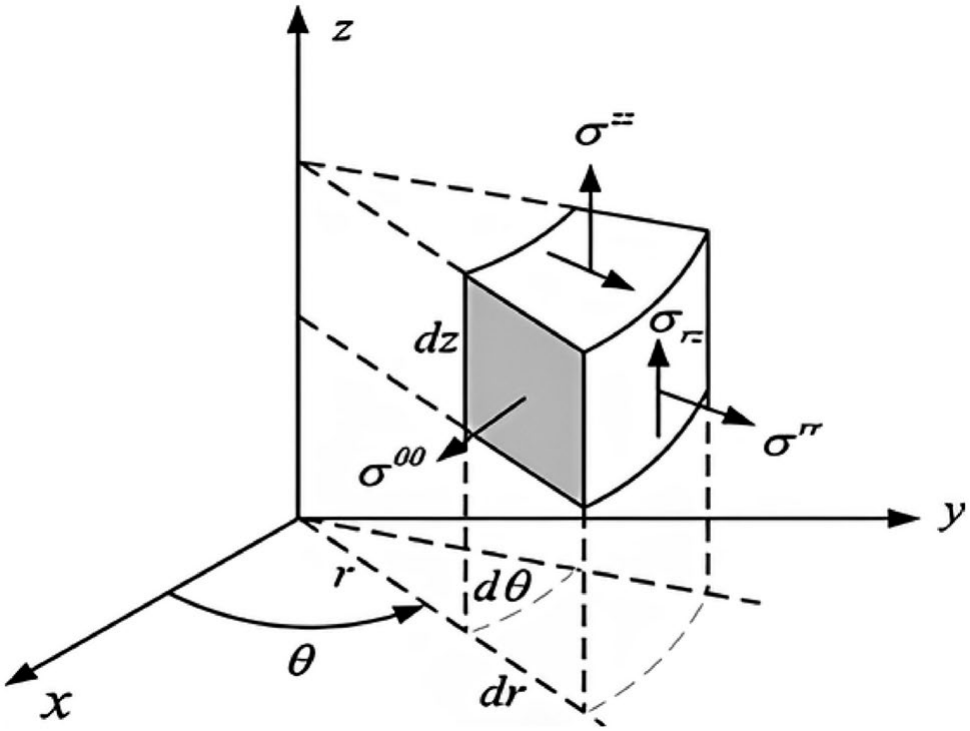
Illustration of an infinitesimal element in cylindrical coordinates with stress components. This diagram depicts a differential element positioned within a cylindrical coordinate system $(r, \theta , z)$ showing the normal and shear stress components $(\sigma _{rr}, \sigma _{\theta \theta }, \sigma _{zz})$ acting on its faces. The element’s orientation demonstrates the relationship between the stresses and the coordinates, essential for analyzing stress in cylindrical bodies under load.

Equations that control mechanical wave propagation are well-known. They are frequently expressed as Partial Differential Equations (PDEs), with the unknown function representing the displacement field of particles within the medium. The SP has an assertive viscoelastic behavior capturing a wide range of experimental data in both time and frequency ranges using power law representations^14^. Torsional waves are mechanical disturbances in which the medium’s particles vibrate perpendicular to the propagation path^19^. They interact with the mechanics of tissue and its spatial variability as mechanical waves. A new family of sensors has recently been patented using torsional waves. Low-frequency sensors that apply torsional shear waves in a substantially solid medium^20^ exemplify this idea. In this new technique, which can produce shear waves of greater intensity, the problem of solid attenuation is resolved, thus enabling the development of new non-invasive tissue characterization techniques. The probe employed in this study was designed to measure the cervix’s shear stiffness. It has been optimized by removing spurious waves (P-waves) by a similar technique to that described in^21^, the excitation energy deposited in the tissue, and its dependence on the applied pressure of the sensor on the tissue. The method’s simplicity ensures a straightforward clinical procedure and a low learning curve for the operator. The construction of a 2D numerical model for torsional wave propagation in skin tissue is described in this section. Geometrically, a simplified skin-like material was modeled as a solid cylinder. Even torsional waves spread from the sample center axisymmetrically^10^. Due to the geometric layout, a cylindrical coordinate system (r,$\theta$,z) can be used. Stresses and strains are illustrated in Fig. 3 to be distributed on an infinitesimal cylindrical element.

**Figure 4:**
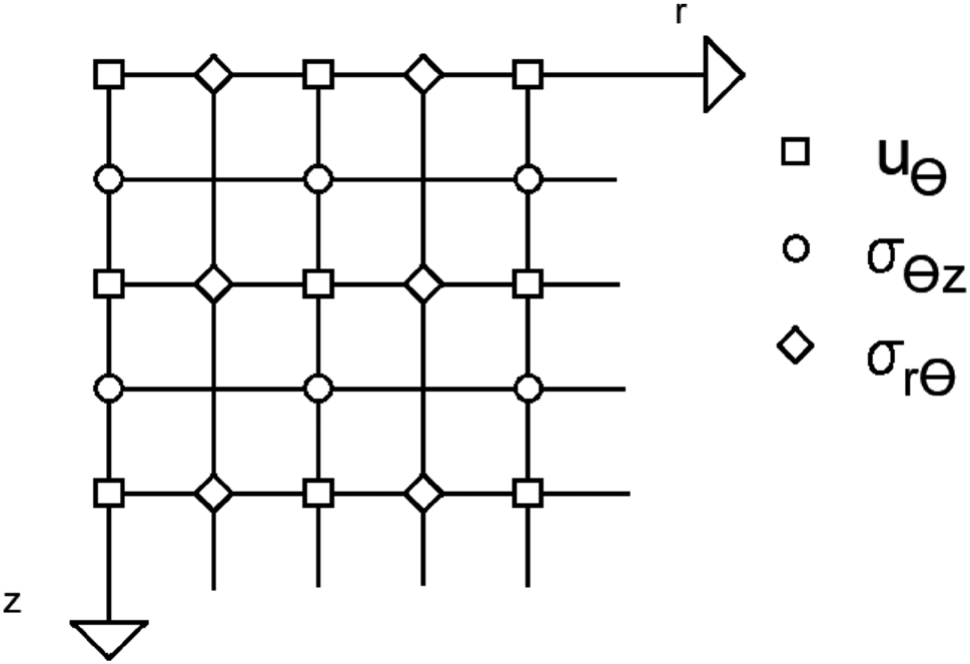
Grid discretization with staggered rows and columns indicating the locations of variables. displacement ($u_{\theta }$) and stresses (${\sigma }_{\theta z }$ ,${\sigma }_{r\theta }$).

Computationally, 3D models are costly. As a result, 2D simplifications are helpful for exploratory research while preserving most of the physics. Absolute axial symmetry was used in this case to reduce the geometry to a two-dimensional case. The wavefront formed by a TWE source is axisymmetrically propagated. This feature and the model’s axisymmetric shape reduce the displacement field to a single component, the angular displacement $u_{\theta }$. Additionally, all the derivatives regarding the variable $\theta$ will be zero. The r-z plane was chosen as the two-dimensional domain to create the wave propagation model (see Fig. 4). The equations governing the propagation of torsional waves through the phantom for the SP model were determined using the equations of motion, the kinematic relationship, and the constitutive equations. The equation system was simplified by excluding all standard components and retaining only the deviatoric (torsional) components. According to Orescanin et al., due to the modest level of normal pressure created by the exciter, in this case, the torsional probe, this simplification did not appear to affect the results^11,22^.

3 $$\begin{aligned} \frac{\partial }{\partial t} \left( \frac{\partial u_\theta }{\partial t}\right)= & {} \frac{\partial \sigma _{r\theta }}{\partial r} + \frac{\partial \sigma _{\theta z}}{\partial z} + \frac{2}{r} \sigma _{r\theta } \end{aligned}$$

4 $$\begin{aligned} \sigma _{r\theta }= & {} K_\alpha \frac{\partial ^\alpha }{\partial t^\alpha } \left( \frac{\partial u_\theta }{\partial r} - \frac{u_\theta }{r}\right) \end{aligned}$$

5 $$\begin{aligned} \sigma _{\theta z}= & {} K_\alpha \frac{\partial ^\alpha }{\partial t^\alpha } \left( \frac{\partial u_\theta }{\partial z}\right) \end{aligned}$$

where K$\alpha$ and $\alpha$ (SP parameters) denote the viscosity and fractional factor, respectively and r, $\theta$, and z denote the cylindrical components, u denotes the particle displacement, and the $\sigma$ is the stress tensor.

### Implementation of the spring pot model

The FDTD method has been widely used to model various physical phenomena in solid materials. But also, for wave propagation^11^ in elastic medium. FDTD solutions can represent a more comprehensive frequency range with a single simulation process and naturally treat nonlinear material properties. Using the formula derived from Taylor series expansions, the FDTD approach was applied to the system of SP equations. Detailed information is available in the general FDTD literature. According to previous works^11,22^, the time-staggering approach utilized in this work involves simultaneously computing all stress, strain, and displacement. With a = i$\Delta$r/2 and b = i$\Delta$z/2 for integers a, b, and space step of discretization r and z, time and space were uniformly sampled see Fig. 4. Table 1 summarizes the parameter values used in the numerical FDTD simulations.

**Table 1:** Parameters used in numerical FDTD simulations.

Grid resolution	Values
Step size $\Delta r$ or $\Delta z$	$3\times 10^{-5}$ m
Time step $\Delta t$	$1\times 10^{-5}$ s
The time of simulation *T*	0.025 s
Frequency step $\Delta f$	40 Hz

After applying space-time discretization to the domain and FDTD expressions to the equations that explain the physical phenomenon of wave propagation in a SP viscoelastic medium, discrete equations are obtained. First, the equations of the problem are split, and the Perfect Matching Layer (PML) parameters are incorporated following the procedure for cylindrical coordinates developed by Liu^23^. The conservation of momentum equation in cylindrical coordinates is split into expressions (6–8). The strain-displacement connection is merged into the SP constitutive law equations reducing the memory needed to compute the method. The resulting equations are then differentiated about time and separated into the expressions (9–11).

6 $$\begin{aligned} a_r \frac{\partial ^2 u_\theta (r)}{\partial t^2} + \omega _r \frac{\partial u_\theta (r)}{\partial t}= & {} \frac{\partial \sigma _{r\theta }}{\partial r} \frac{1}{\rho } \frac{\partial r}{\partial r} \end{aligned}$$

7 $$\begin{aligned} A_r \frac{\partial ^2 u_\theta (\theta )}{\partial t^2} + \Omega _r \frac{\partial u_\theta (\theta )}{\partial t}= & {} \frac{2\sigma _{r\theta }}{\rho } \end{aligned}$$

8 $$\begin{aligned} a_z \frac{\partial ^2 u_\theta (z)}{\partial t^2} + \omega _z \frac{\partial u_\theta (z)}{\partial t}= & {} \frac{\partial \sigma _{\theta z}}{\partial z} \frac{1}{\rho } \frac{\partial z}{\partial z} \end{aligned}$$

9 $$\begin{aligned} a_r \frac{\partial \sigma _{r\theta }(r)}{\partial t} + \omega _r \sigma _{r\theta }(r)= & {} K_\alpha \frac{\partial ^{\alpha +1}}{\partial t^{\alpha +1}} \left( \frac{\partial u_\theta }{\partial r}\right) \end{aligned}$$

10 $$\begin{aligned} A_r \frac{\partial \sigma _{r\theta }(\theta )}{\partial t} + \Omega _r \sigma _{r\theta }(\theta )= & {} -K_\alpha \frac{\partial ^{\alpha +1} u_\theta }{\partial t^{\alpha +1}} \end{aligned}$$

11 $$\begin{aligned} a_z \frac{\partial \sigma _{\theta z}(z)}{\partial t} + \omega _z \sigma _{\theta z}(z)= & {} K_\alpha \frac{\partial ^{\alpha +1}}{\partial t^{\alpha +1}} \left( \frac{\partial u_\theta }{\partial z}\right) \end{aligned}$$

where $u_\theta$ = $u_\theta (r)$ +$u_\theta (\theta )$ +$u_\theta (z)$ according to the notation employed by Ref.^23^. The PML variables described by Liu (1999) include $a_r$, $A_r$, and $a_z$, where $a_r = a_z = 1$, and $A_r = \frac{1}{r}$. The absorbing parameters are denoted by $\omega _r$, $\Omega _r$, and $\omega _z$.

### Numerical model setup

**Figure 5:**
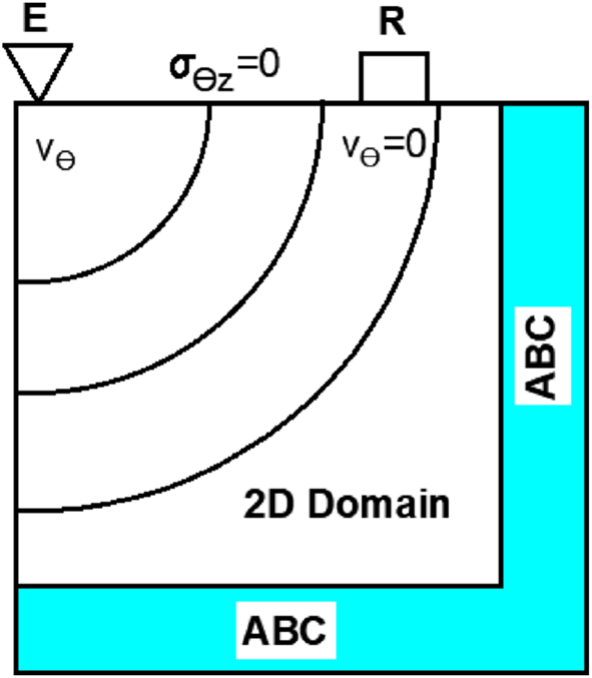
The spatial distribution of the model boundary conditions. A two-dimensional domain is bordered by absorbing boundary conditions, excitation, reception ($v_{\theta }$ = 0), and free surface conditions.

The dimension ranges corresponded to the size of normal human skin tissue under pathological disorders. Males have a range of skin thickness from 0.6 mm to 3.3 mm, while females have a range of skin thickness from 1.3 to 3.1 mm^24^. After reviewing the literature on these tumors, even the form, such as a circular mark with a diameter of 3 to 13 mm, was consistent^2^. These tumors are less than 1 mm thick in the early stage (target stage)^25^. For our case study, we chose a tumor that resembled a 9 mm in diameter and 1 mm in thickness circular area. Figure 5 depicts the result of applying this form to our axisymmetric model. The TWE probe was not modeled physically. Instead, the excitation displacement signal was applied directly to the mesh elements of the model’s surface where the probe would be inserted. Similarly, instead of modeling the array of sensors, the displacement values at the mesh elements in physical contact with the sensors’ locations were recorded. see Fig. 5. The Parallel Computing Toolbox was used to implement the FDTD wave propagation model in MATLAB®(Release 2018a) (Release 2018a, MathWorks, Natick, United States). Figure 5, illustrates the dimensions of the emitter, receiver, emitter-receiver, and receiver-ABC. The boundary conditions for the two-dimensional space were as follows (Fig. 5): the boundary conditions of the problem were the excitation source at specific points on the sample’s surface: the absence of shear stress on the surface as a free boundary, and the absence of velocity in the grid point on the reception location due to the pressure applied between the receiver and the sample^22^.

$$\begin{aligned} \sigma _{\theta z}(r,0,t_n)&= 0 \\ v_\theta (r_{\text {reception}},0,t_n)&= 0 \end{aligned}$$

**Figure 6:**
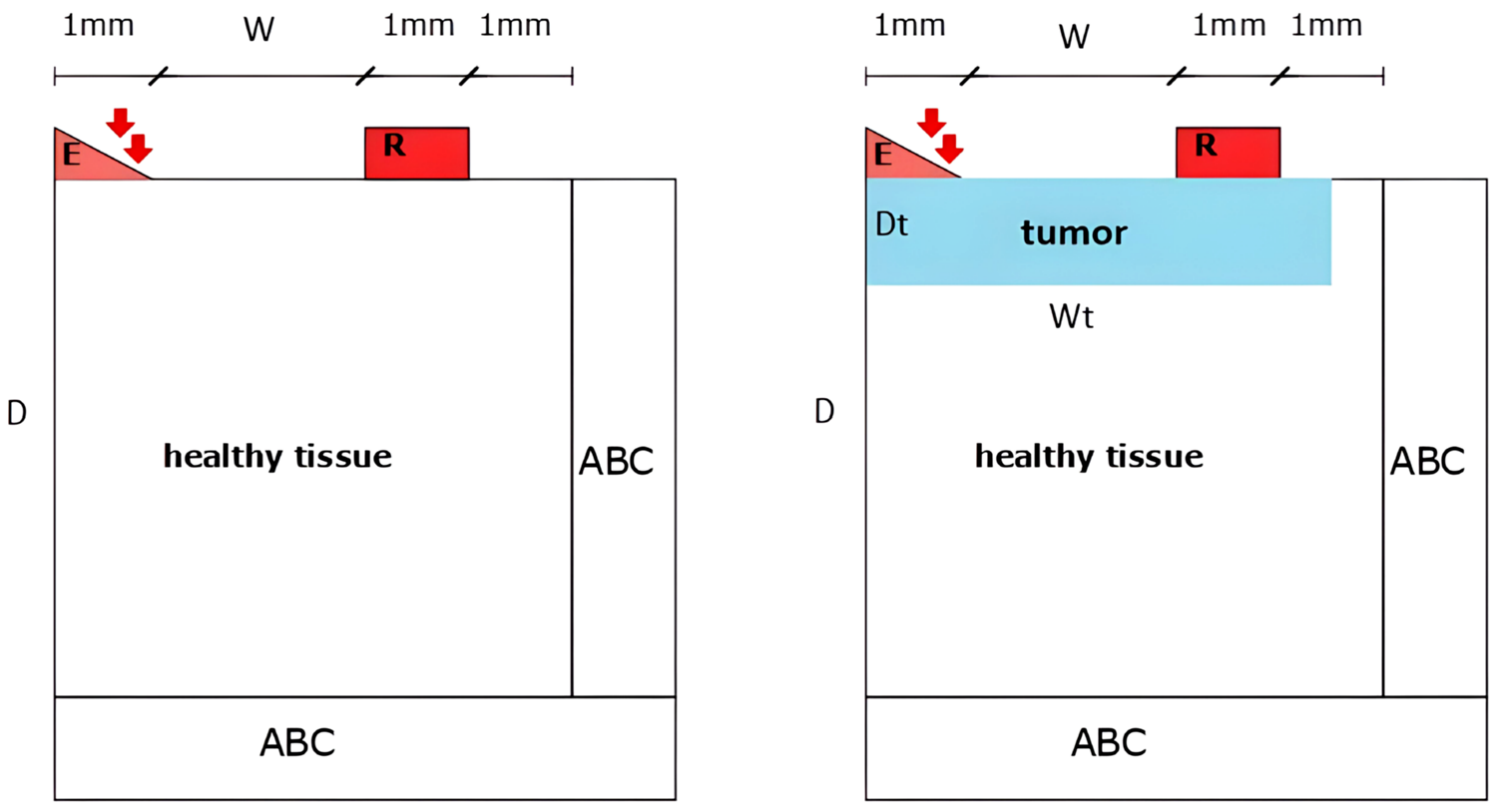
The first scenario involves healthy tissue by itself, whereas the second case involves healthy tissue with a tumor of varying thicknesses.

The shear wave velocity of the model can be determined directly from the time of flight^10^. The examination of the resulting signal enables the determination of the time of flight as the difference between the starting times of the received and excitation signals^10^. However, certain simplifications must be made to determine the start of the signal. Several approaches to signal normalization have been described in the literature. In this work, the signal is normalized to the first maximum, and the start is calculated from this maximum using a reference value (typically 5% or 0.05). Now, we can obtain the shear wave velocity at any frequency by determining the time of flight and the distance between the emitter and the receiver. Then, we repeat the previous method for a range of frequencies to plot the dispersion curves for the SP model in various scenarios. We examined two scenarios. In the first case, we analyzed only healthy tissue; in the second case, we studied healthy tissue in combination with a tumor with different thicknesses. See Fig. 6.

The next stage is to evaluate the SP model using different metrics to determine their sensitivity to mechanical parameters and geometric characteristics (tumor depth, i.e., 1, 2, 3, 4 mm, and tumor width) (see Table 2). Analogous skin tissue studies determined the search ranges for the parameter’s stiffness, viscosity, and alpha for elasticity^26,27^ and for viscosity^28^. The American Joint Committee on Cancer (AJCC) uses Breslow thickness and Clark level in its staging system for malignant skin cancers. Breslow thickness measures the size of melanoma growth in terms of how deep it has penetrated into the epidermis. The greater the Breslow thickness, the greater the risk of metastases and the poorer the patient’s prognosis^25^. According to the American cancer society, the tumor’s size is greater than 6 mm in diameter—about the size of a pencil eraser - but melanomas can occasionally be smaller (between 3 and 13 mm)^2^.

**Table 2:** The dimension range utilized in the geometric study.

The dimension	The range of study
Depth of model (D)	0.1–4 mm
Width of the model (W)	0.1–3 mm
Depth of tumor (Dt)	0.1–4 mm
Width of tumor (Wt)	0.1–6 mm

### Experimental validation

Validation of computational models can be accomplished through the use of various methods, including experimental investigation. Experimentation verifies the model ability to reproduce the physical occurrence objectively. The model is validated when simulation results and experimental data reach a predefined agreement threshold. Specific characteristics of physical phenomena that can be compared to model simulations can be measured using well-established alternative approaches. This part describes the experimental work to validate the TWE- SP wave propagation model described in the previous section.

#### Phantom preparation

In the literature, various tissue-mimicking materials and phantoms are mentioned as utilizing various materials and preparation procedures to represent various biological systems. In ultrasonography phantoms, mixtures of Agar and gelatin, polyacrylamide gel, paraffin-gel waxes, polyvinyl chloride (PVC), and polyvinyl alcohol (PVA) were utilized^29–37^. Both Agar and gelatin have exceptional acoustic qualities for ultrasound (US) elastography, making them useful for both magnetic resonance (MR) and ultrasound^38,39^. Combinations of Agar and gelatin are frequently used to replicate soft tissues’ acoustic and elastic properties^40,41^. After examining several components and production techniques, the ingredients in Table 3 were proposed. Phantoms are fabricated using gelatin and agar as their base components. Xanthan gum functions as a suspending agent, whereas cellulose acts as speckles. Castor Oil is also used to modify the viscosity of tissue phantoms. To obtain the appropriate mechanical qualities, varying amounts of castor oil were added to the tissue phantom. Lastly, sodium dodecyl sulfate (a surfactant) was added to improve the mixing of the oil into water. Phantoms contain various gelatin, Agar, and oil concentrations to simulate the elasticity and viscosity of skin tissue. Considering that skin cancer is constituted of tumor and normal layers, the phantoms were constructed with two layers in consideration. The percentages of gelatin, agar, and oil, as well as the thickness of the first layer (simulating the cancerous layer of the skin), were determined to imitate the viscoelastic qualities of skin tissue based on findings from the scientific literature. Eight different phantoms have been created for this purpose. Variable ingredients such as gelatin, Agar, oil, and different layer thickness are the only parameters that affect the SP model’s parameters; other ingredients were held constant. The design of our phantoms aimed to faithfully replicate the distinct mechanical properties of the healthy and unhealthy tissues, as reported in the literature. To achieve this, we tailored the composition of each layer in our phantoms to reflect these characteristics. For instance, the cancerous layer was formulated with higher percentages of gelatin and agar to simulate its stiffness relative to the healthy tissue. This meticulous approach to phantom composition was crucial for accurately simulating the mechanical properties of each skin layer and observing the resulting wave behavior.

**Figure 7:**
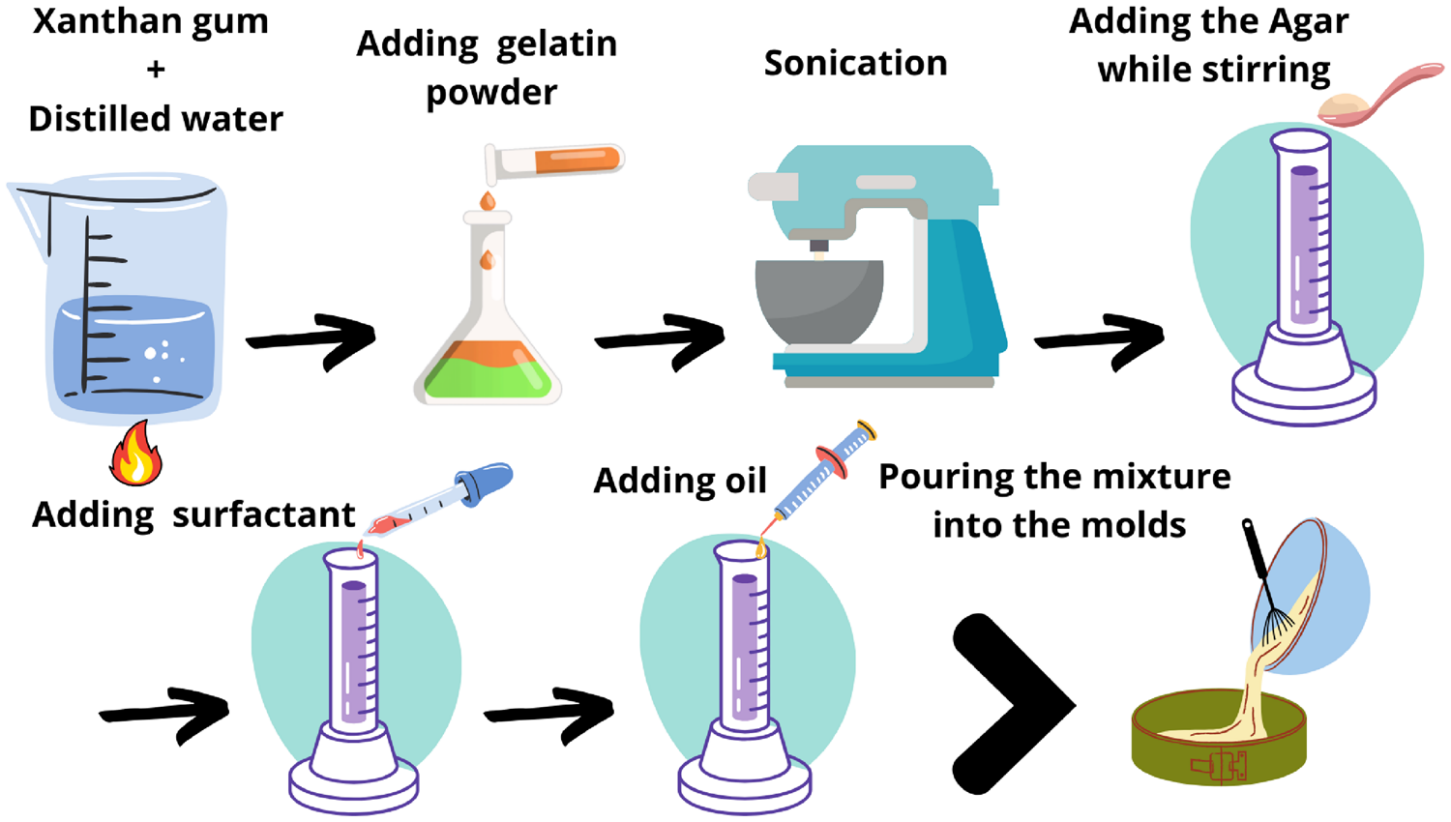
Navigating the phantom process: a visual roadmap.

The objective is to alter the parameters of each reconstructed phantom and verify that the Probabilistic Inverse Problem (PIP) can reconstruct them. Table 4 provides information regarding the percentages of gelatin, agar, and oil and the thickness of the two layers. The approach for creating the phantoms required for measurements with the TWE technique is outlined below (see Fig. 7).

1. Weigh and prepare each of the components listed in Table 3.
2. Heat water to (70 $^\circ$C).
3. Add the xanthan gum to the distilled water and mix for 5 min.
4. Gradually add the gelatin powder to the solution and mix for 5 min.
5. Sonication disrupts compounds using sound waves. The conversion of an electrical signal into a vibration can mix solutions, dissolve particles in liquids, and remove dissolved gases from liquids. Put the mixture through five cycles of the Sonication (55-s pulse, 5-s rest) with frequency of 20 kHz and amplitude of 65%.
6. Place the mixture on a magnetic stirrer and add the Agar while stirring.
7. Mix for a further 5 min after adding the surfactant.
8. Add the oil to the solution and continue mixing.
9. Waiting until the mixture’s temperature decreases to between 40$^\circ$ and 50$^\circ$ before adding cellulose (mixing it well with the mixture and keeping it from going down) and waiting 15 min while mixing correctly.
10. Pour the mixture into the molds (7 $\times$ 7 cm in dimension) until the second layer reaches the desired thickness.
11. Repeat steps 1 through 10 using the ingredients required to create the first layer while the first batch is reaching to the room temprature.
12. Pour the second batch (first layer) carefully over the first batch once both batches have reached a temperature of between 37 and 38 $^\circ$C.
13. Before putting the phantom in the refrigerator, allow it to solidify at room temperature for 2 h.
14. Remove the phantom from the fridge and let it reach to the room temperature for 6 h.

**Table 3:** Components of the tissue-replicating phantoms gelatin solution. Each batch had a different ratio of gelatin, agar, and oil.

Ingredient	The percentage	Supplier, type
Gelatine	[5%, 7.5%, 10%, 15%]	Sigma Aldrich, Laboratory chemicals
Agar	1% and 2%	Thermofisher S, Agar, pure, powder
Cellulose	1%	Sigma Aldrich, Type 50, 50 $\upmu$m
Xanthan gum	0.5%	E415
Castor Oil	[5%, 10%, 15%]	PraNaturals, cold pressed castor oil
Sodium dodecyl	0.1%	Sigma Aldrich, ACS reagent, $\ge$ 99%
H2O (100 mL)	100 mL	Laboratory distilled water

**Table 4:** Gelatin, agar, and oil w/w percentages, as well as the thickness of the two layers, for each of the twelve phantoms.

Phantom	Layer	Gelatin %	Oil %	Agar %	Thick (mm)
1	First layer	7.5	5	1	1
	Second layer	5	5	1	4.6
2	First layer	7.5	5	1	3
	Second layer	5	5	1	4.6
3	First layer	10	5	1	1
	Second layer	5	5	1	4.6
4	First layer	10	5	1	3
	Second layer	5	5	1	4.6
5	First layer	15	5	1	1
	Second layer	10	5	1	4.6
6	First layer	15	5	1	3
	Second layer	10	5	1	4.6
7	First layer	15	5	1	4
	Second layer	10	5	1	4.6
8	First layer	15	5	2	1
	Second layer	10	5	1	4.6
9	First layer	15	5	2	3
	Second layer	10	5	1	4.6
10	First layer	15	5	2	4
	Second layer	10	5	1	4.6
11	First layer	15	10	2	4
	Second layer	10	5	1	4.6
12	First layer	15	15	2	4
	Second layer	10	5	1	4.6

Optimization was an integral part of our phantom development, focused on closely replicating the mechanical and acoustic properties of soft tissue. Key parameters considered included:

- **Base Material Selection:** Gelatin was chosen due to its similarity to soft tissue mechanics, ease of preparation, and cost-effectiveness, allowing for the creation of multiple phantoms. Viscous agents like Xanthan gum and castor oil were added to enhance viscosity.
- **Acoustic Property Matching:** Hydrogel compositions were adjusted to emulate the speed of sound, attenuation, and absorption characteristics of soft tissue, facilitating realistic ultrasound simulation. The TWE and TW-UI tests, conducted at low frequencies up to 1kHz, were crucial for this aspect.
- **Scattering Characteristics:** Agar and cellulose concentrations were varied as scattering agents to produce realistic ultrasound images. This approach helped in simulating the tissue’s speckle patterns during TW-UI testing.
- **Phantom Homogeneity and Stability:** Each phantom underwent a freezing-thaw cycle and was equilibrated to room temperature before testing, ensuring any differences in results were due to material concentrations rather than external variables.

This comprehensive optimization strategy was pivotal in achieving phantoms that not only simulate the physical characteristics of soft tissue but also provide a reliable platform for evaluating our TWE device and methodology.

#### TWE characterization of the phantoms

**Figure 8:**
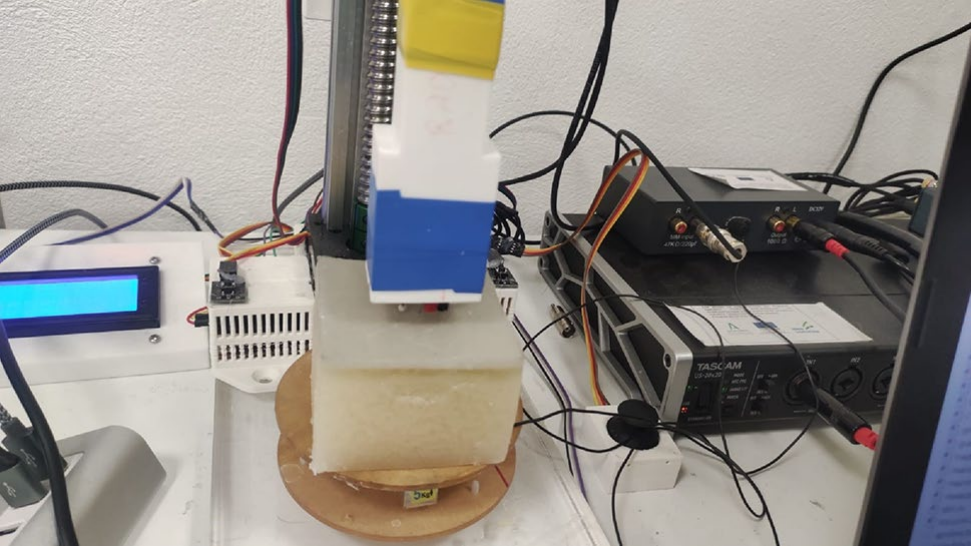
Experimental Setup for Torsional Wave Elastography. This photograph illustrates the experimental configuration, showcasing the phantom material placed beneath the torsional wave sensor. The setup includes the hardware for wave generation and reception, connected to a laptop running analytical software, which displays a typical waveform acquired during testing.

After the phantoms reached to the laboratory temperature (22 ± 1 Co), they were examined, and a scale was used to quantify the applied pressure during the measurement (see Fig. 8). The Ultrasonics Lab team at the University of Granada constructed and designed a probe capable of generating, receiving, and interpreting torsional waves, which was used to assess each of the twelve bi-layer phantoms in three distinct areas^9,10,21^. The propagating torsional wave is a one cycle sine-burst with a frequency of 1000 Hz. The torsional sensor gets a voltage signal representing the interaction with the various phantom layers.

#### Validation of the TWE using ultrafast ultrasound imaging

**Figure 9:**
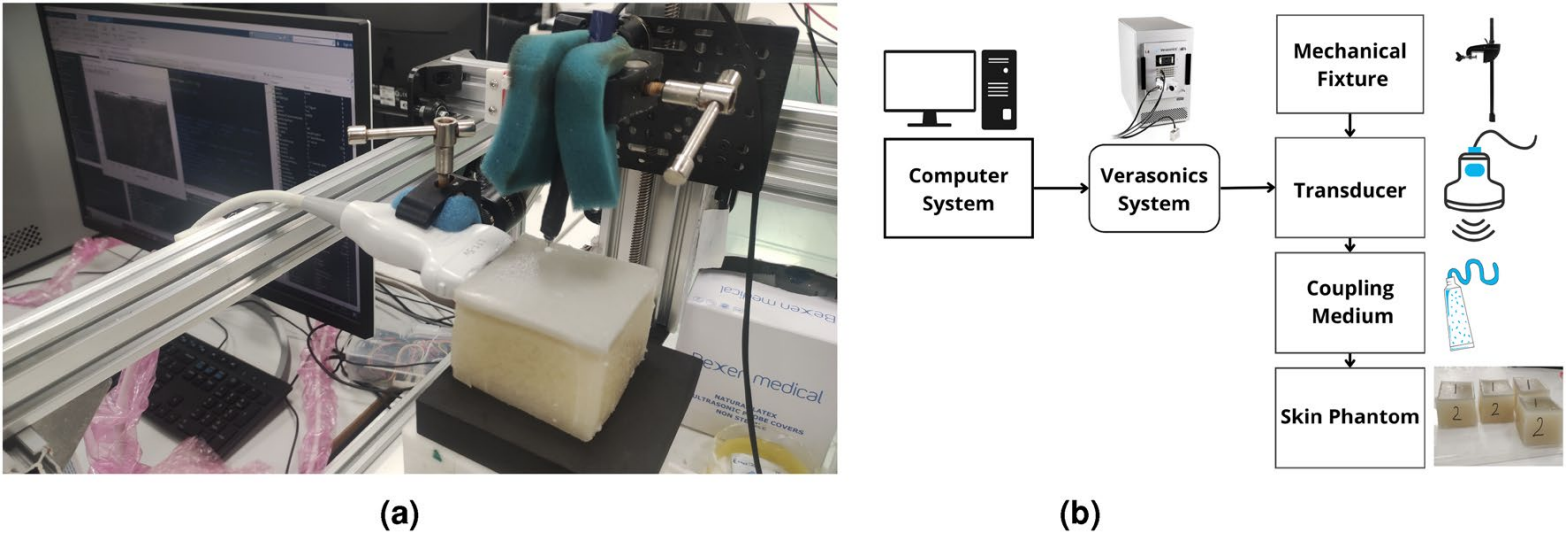
Imaging experimental setup. This figure shows the setup used for ultrafast imaging, with key components such as the transducer, phantom material, and the imaging system’s control unit highlighted. The setup is positioned to capture high-resolution images of shear wave propagation in the phantom.

The Verasonics Vantage System was employed to validate the experimental work, leveraging its capabilities as an advanced ultrasound imaging and research system. This system is renowned for its high performance and versatility across ultrasound imaging systems, research applications, and software development kits. Widely recognized for its application in academic, preclinical and clinical research, as well as industrial testing and inspection, the Verasonics systems offer a broad utility spectrum^42^. For our study, the Torsional Wave Ultrafast Imaging (TW-UI) technique was introduced, using an external wave generator to produce a single cycle of a sinusoidal torsional wave with a strategic excitation frequency of 1 kHz and an amplitude of 500 mV. This specific frequency was chosen to facilitate a meaningful comparison with the TWE technique, given its capability to characterize the medium’s mechanical properties at frequencies below 1kHz. The propagated torsional wave’s behavior was tracked and captured utilizing the Verasonics L11-5v transducer, a 128-element linear array transducer with a central frequency of 7.5 MHz, to observe the wave as it moved through various ROIs within the phantoms (see Fig. 9). The transducer plays a crucial role in converting the reflected ultrasonic waves from the objects into electrical impulses, allowing for the extraction of detailed properties of the emitted wave in the phantoms^43^. This sophisticated integration of the TW-UI technique with the Verasonics system underscores our experimental setup’s innovation, providing a robust platform for the precise analysis of mechanical wave propagation and the viscoelastic properties of tissue-mimicking phantoms.

.

### Reconstructing skin tissue mechanical properties using probabilistic inverse problem

The concept for constructing viscoelastic parameters is based on the effect of viscoelasticity on the wave velocity within soft tissue. In this instance, the cause can be determined from the effect; therefore, our problem is an inversion of a forward problem. Moreover, to obtain viscoelastic parameters, we must solve the inverse problem. To overcome this difficulty, we will use a novel approach based on a probabilistic inverse problem and a logical inference framework developed by Rus et al^44,45^ to determine the most probable physical model parameters for a viscoelastic material. The information-theoretic inverse problem paradigm is then used to describe the parametrization procedure, the operation with discrete signal observation data, and its application to probabilistic parameter optimization.

The information-theoretic inverse problem framework explains the parametrization process, the operation with discrete observation data of signals, and two crucial extensions: hypothesis testing H and parameter optimization M. To determine the efficacy of the PIP approach, we solve the following inverse problem: The outputs are the viscoelastic constitutive mechanical properties of the assessed soft tissue^44–53^. The values of the parameter search range have been determined using scientific evidence; see Table 5.

**Table 5:** Search range of the spring pot model parameters.

Parameter	The range of study
Viscosity	0–30 Pa s
Alpha	0–1
Thickness	1–4 mm
Frequency	400–1000 Hz

## Results

### Validation of torsional waves sensor measurement from time-of-flight using torsional wave-ultrafast imaging

**Figure 10:**
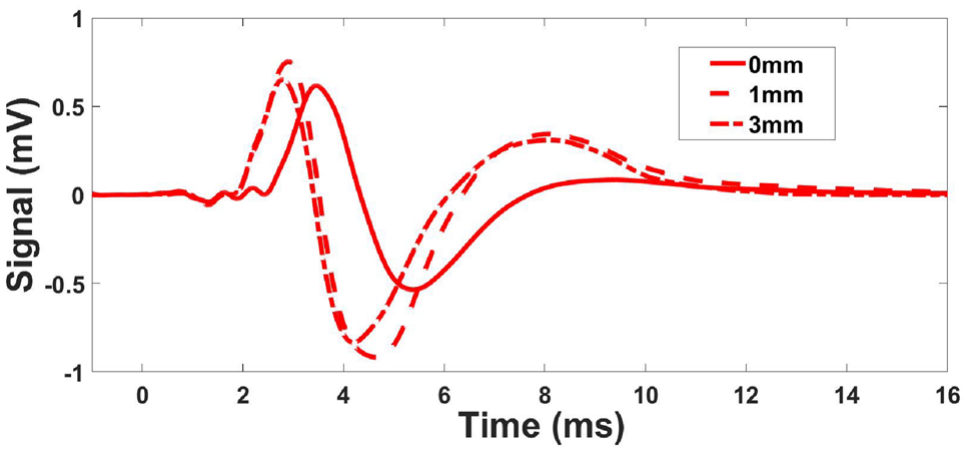
Examples of experimental signals at 500 Hz with varying first layer thickness for phantoms 1 and 2.

**Figure 11:**
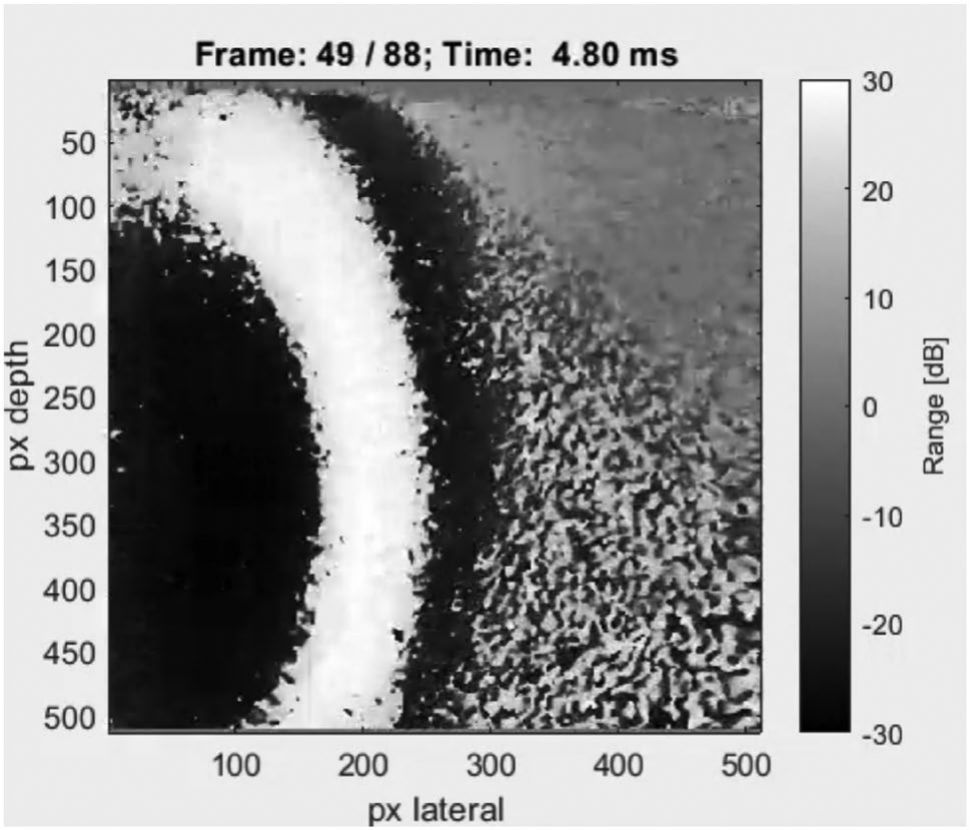
Using the Verasonic’s vantage system, the image depicts the propagation of torsion waves inside a phantom.

Various experimental signals generated by the TWE sensor designed and fabricated by the Ultrasounics Lab team are depicted in Fig. 10. This figure depicts the signals for a group of phantoms at 500 Hz with different thicknesses for the first layer. Measured signals were utilized by the PIP to determine the properties of the tissue in the next section. Figure 11 shows the propagated torsional wave tracked by using the L11-5v transducer, Verasonic vantage system. The velocity measurements for phantoms are shown in Table 6 using two methods: first, by TWE sensor, and second, by Ultrafast Imaging carried out by the Verasonics Research System. Shear wave speeds obtained after analyzing the data of two methods using the time of flight approach as the difference between the starting times of the received and excitation signals for each single-layer phantom (see “Numerical model setup”).

**Table 6:** Velocity measurements for phantoms made using two methods: first, by TWE sensor, and second, by ultrafast imaging.

Phantoms	Torsion velocity (m/s)	Ultrafast imaging (m/s)
1	2.434 ± 0.121	2.641 ± 0.012
2	2.494 ± 0.120	2.680 ± 0.019
3	3.904 ± 0.351	3.797 ± 0.074
4	3.931 ± 0.325	3.831 ± 0.225
5	4.379 ± 0.343	4.395 ± 0.089
6	4.45 ± 0.430	4.482 ± 0.053
7	4.191 ± 0.362	4.230 ± 0.217
8	4.563 ± 0.309	4.781 ± 0.118
9	4.54 ± 0.265	5.013 ± 0.016
10	4.359 ± 0.267	4.892 ± 0.198
11	4.327 ± 0.349	4.368 ± 0.066
12	4.332 ± 0.329	4.414 ± 0.077

### Phantom’s properties validation range

**Figure 12:**
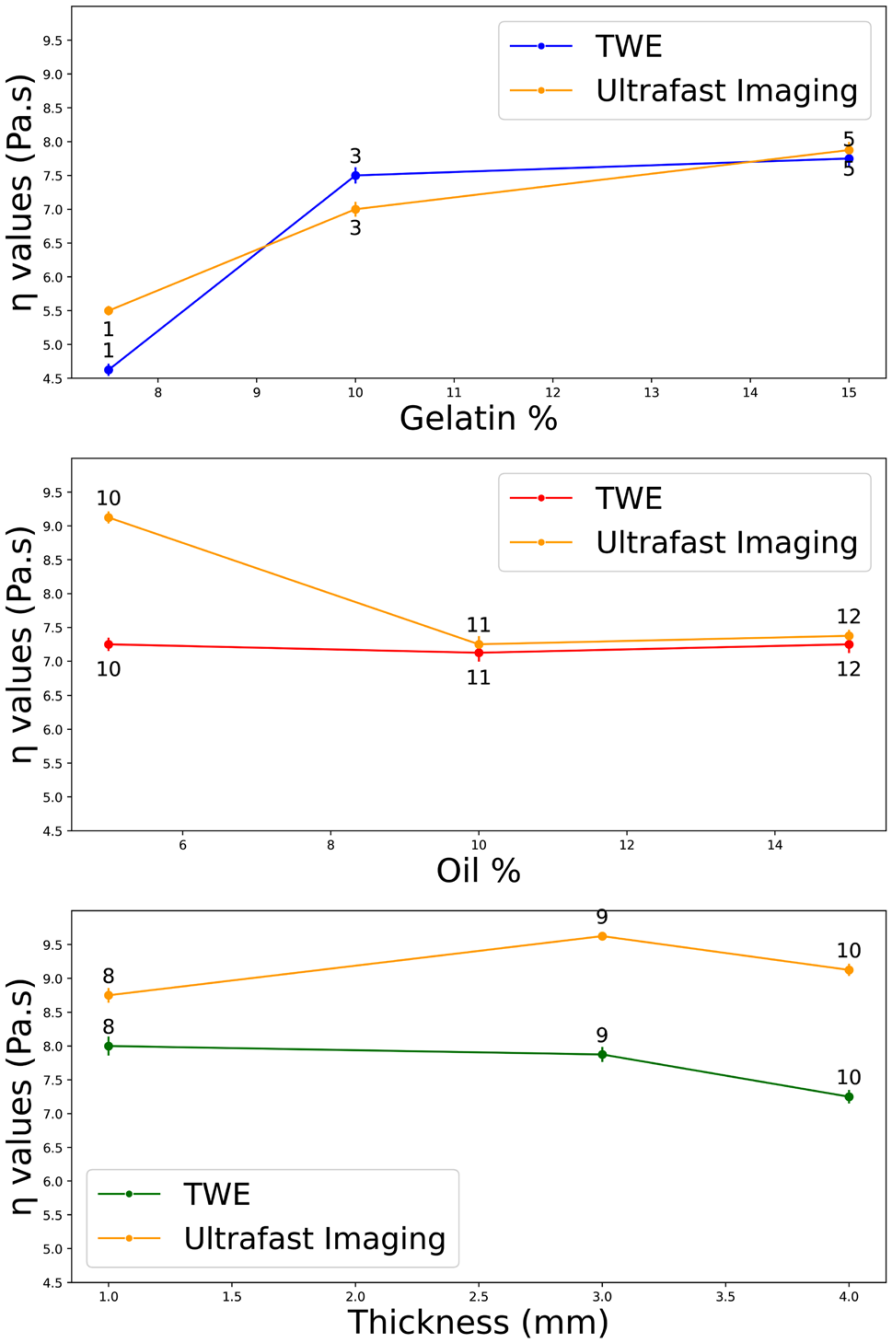
Effect of gelatin, oil percentages, and thickness on $\eta$1 in the phantoms for the two techniques, torsion probe and ultrafast imaging approach (Numbers on points represent the Phantoms).

**Figure 13:**
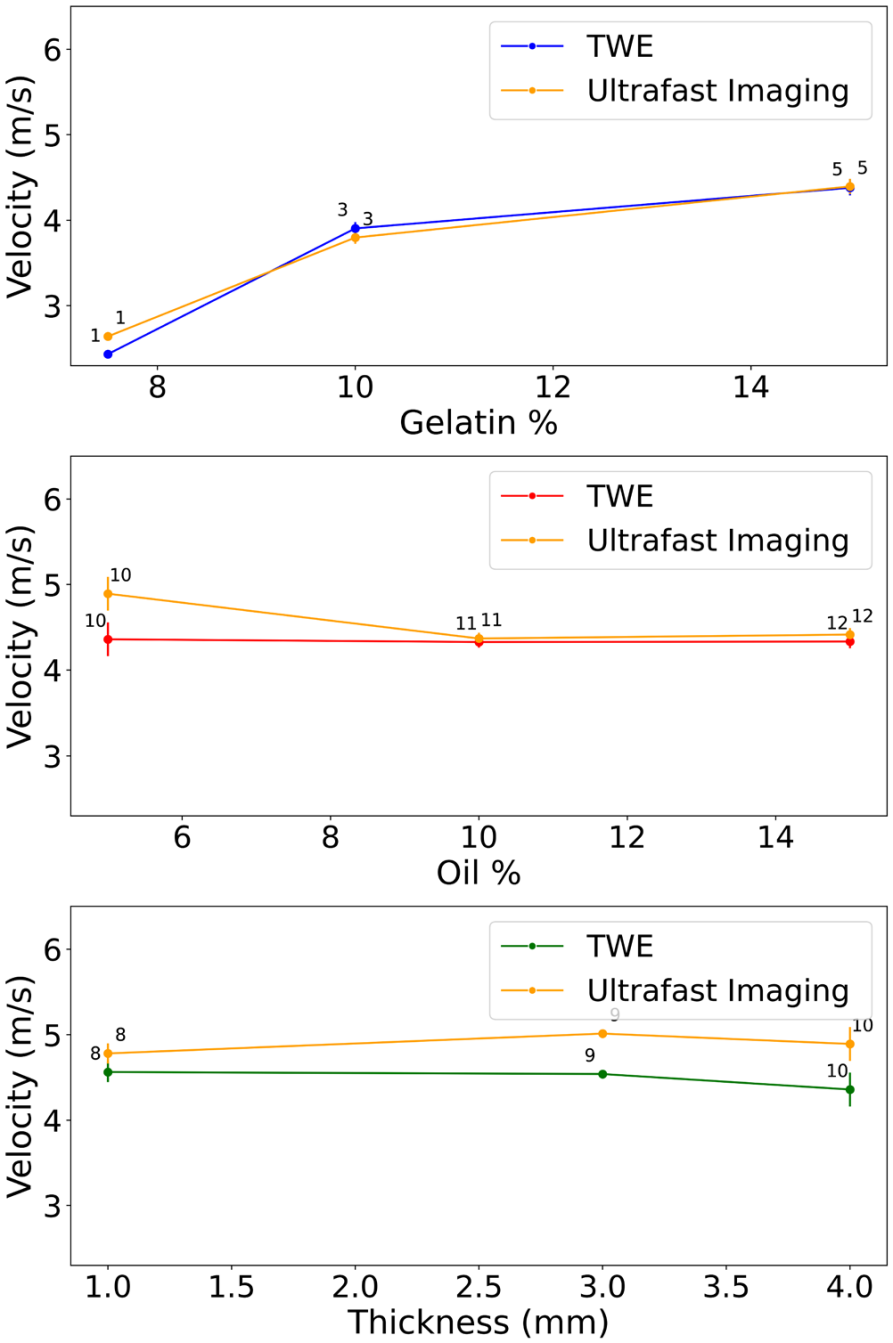
Effect of gelatin, oil percentages and thickness on torsional wave velocity in the phantoms for the two techniques, TWE and ultrafast imaging approach (numbers on points represent the phantoms).

After set of data analysis, the ultrafast Imaging and TWE technique SP parameters are provided in Table 7, obtaining wave velocity as a function of frequency for each technique and fitting curves using Eqs. (1) and (2). In our study, each measurement on the phantoms was repeated three times to ensure reproducibility and reliability. This practice is in alignment with standard scientific methods where repeated measures are utilized to confirm result consistency. The low variability observed across these repetitions substantiates the precision of our methodology. Furthermore, the choice of three repeats represents a balance between statistical reliability and experimental feasibility, considering the time and resources available. Effect of gelatin, oil percentages, and thickness on $\eta$1 in the phantoms for the two methods,torsion probe and ultrafast imaging technique, are shown in Fig. 12. Figure 13 shows the effect of material percentage on torsional wave velocity as represented by phantoms.

**Table 7:** TWE versus ultrafast imaging carried out by the verasonics research system spring pot parameters for each phantom.

	TWE		Ultrafast imaging	
	$\alpha _1$	$\eta _1$ (Pa s)	$\alpha _1$	$\eta _1$ (Pa s)
Ph 1	0.80 ± 0.003	4.625 ± 0.09	0.80 ± 0.002	5.50 ± 0.07
Ph 2	0.80 ± 0.002	4.875 ± 0.08	0.80 ± 0.002	5.625 ± 0.06
Ph 3	0.85 ± 0.006	7.5 ± ± 0.12	0.85 ± 0.005	7.0 ± 0.11
Ph 4	0.85 ± 0.007	7.5 ± 0.14	0.85 ± 0.004	6.75 ± 0.09
Ph 5	0.85 ± 0.006	7.75 ± 0.13	0.85 ± 0.006	7.8750 ± 0.12
Ph 6	0.85 ± 0.009	7.8750 ± 0.15	0.85 ± 0.003	8.0 ± 0.14
Ph 7	0.85 ± 0.008	7.625 ± 0.11	0.85 ± ± ± 0.005	7.625 ± 0.16
Ph 8	0.875 ± 0.006	8.0 ± 0.14	0.8750 ± 0.002	8.750 ± 0.11
Ph 9	0.875 ± ± 0.004	7.875 ± 0.11	0.8750 ± 0.005	9.625 ± 0.06
Ph 10	0.875 ± 0.003	7.250 ± 0.10	0.8750 ± 0.005	9.125 ± 0.09
Ph 11	0.875 ± 0.001	7.125 ± 0.13	0.8750 ± 0.002	7.250 ± 0.12
Ph 12	0.875 ± 0.004	7.25 ± 0.13	0.8750 ± 0.004	7.375 ± 0.09

Values for the mean and standard deviation were calculated from three distinct measurements.

### Reconstructing spring-pot FDTD parameters for multi-layered tissue characterization from TWE measurements

**Figure 14:**
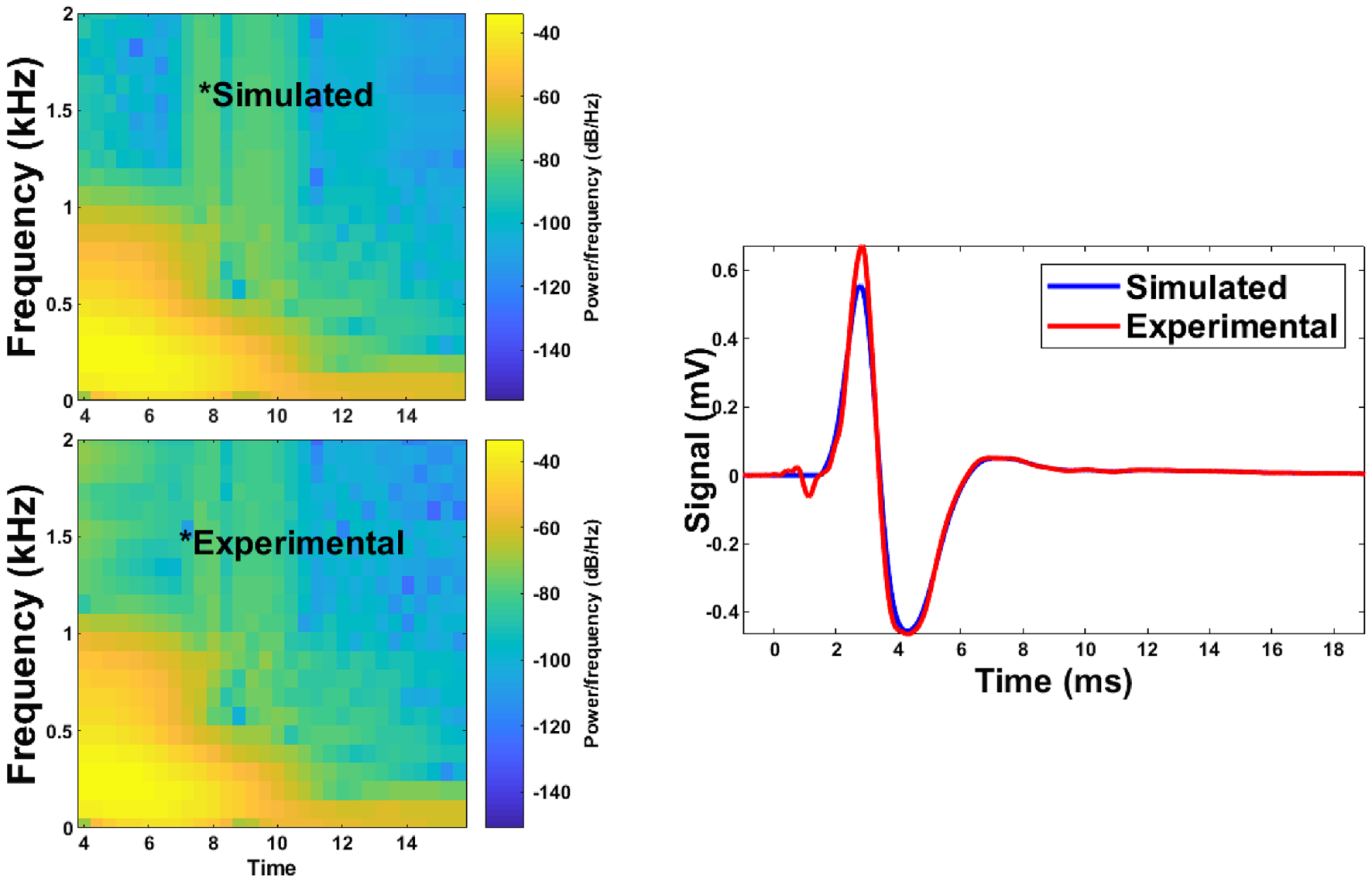
An example of fitting experimental and simulated signals using the spring-pot model in the time domain for Phantom 1.

**Figure 15:**
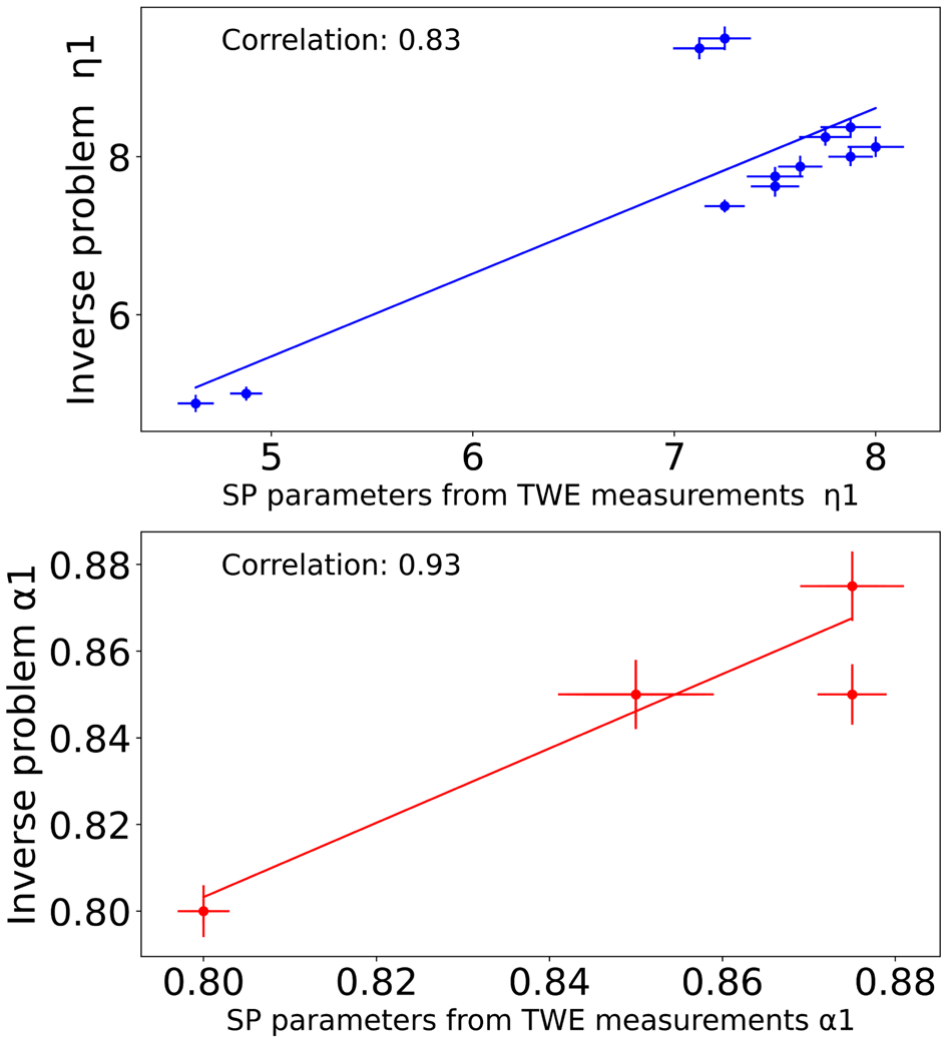
The correlation between the SP parameters reconstruction from TWE measurements for the single-layer phantom, which uses Eqs. (1) and (2), and the inverse problem method for reconstructing the parameters.

After solving the probabilistic inverse problem, which consists mainly of comparing the experimental signals (obtained through TWE measurements) with those derived from the SP-FDTD model (“Numerical model setup”), the estimated parameters are listed in Table 8. After solving the PIP, the first layer shear viscosity, alpha, and thickness were recreated. Three independent measurements for each specimen were used to calculate the mean and standard deviation values. Figure 14 shows how Pearson correlation, dynamic time warping (DTW), and time-frequency representation can fit experimental and synthetic signals for Phantom 1. Shear wave speeds obtained after analyzing the TWE data as a function of frequency are used to reconstruct the SP parameters by Eqs. (1) and (2). The SP parameters for each fitted curve are shown in Fig. 15 for the single-layer phantom. The mean and standard deviation values were estimated from three independent measurements. Finally, the correlation between SP parameters reconstruction from TWE measurements and the inverse problem method for reconstructing the SP parameters is shown in Fig. 15. $\eta$1 and $\alpha$1 have Pearson correlations of 0.83 an0.93, respectively.

**Table 8:** Reconstruction of spring pot parameters from TWE.

	First layer		Thickness (mm)	Second layer	
	$\alpha _1$	$\eta _1$ (Pa s)		$\alpha _2$	$\eta _2$ (Pa s)
Ph 1	0.8 ± 0.006	4.875 ± 0.11	1	0.75 ± 0.004	5.00 ± 0.12
Ph 2	0.8 ± 0.003	5.00 ± 0.09	3	0.75 ± 0.002	4.95 ± 0.06
Ph 3	0.85 ± 0.007	7.625 ± 0.13	1	0.75 ± 0.008	5.25 ± 0.11
Ph 4	0.85 ± 0.008	7.750 ± 0.12	3	0.75 ± 0.007	6.00 ± 0.13
Ph 5	0.85 ± 0.005	8.25 ± 0.11	1	0.875 ± 0.009	6.25 ± 0.14
Ph 6	0.85 ± 0.004	8.374 ± 0.12	3	0.875 ± 0.008	6.5 ± 0.15
Ph 7	0.85 ± 0.008	7.875 ± 0.14	4	0.875 ± 0.007	7.00 ± 0.12
Ph 8	0.875 ± 0.008	8.125 ± 0.13	1	0.875 ± 0.006	6.50 ± 0.11
Ph 9	0.875 ± 0.002	8.00 ± 0.12	3	0.875 ± 0.004	6.50 ± 0.14
Ph 10	0.875 ± 0.001	7.375 ± 0.08	4	0.875 ± 0.005	7.00 ± 0.11
Ph 11	0.85 ± 0.007	9.375 ± 0.14	4	0.8 ± 0.009	6.625 ± 0.16
Ph 12	0.85 ± 0.006	9.5 ± 0.15	4	0.8 ± 0.008	6.5 ± 0.19

The shear viscosity and alpha of the first and second layers and the thickness of the first layer for each of the twelve phantoms.

## Discussion

Characterizing soft tissue’s viscoelastic characteristics is critical in developing numerous medical applications based on elastography imaging. Validation studies are required to satisfy the growing interest in elastography techniques for assessing viscoelastic properties. Rheological methods are currently the gold standard for measuring the viscoelastic characteristics of soft tissues. These approaches, however, are restricted to in-vitro and ex-vivo samples. For example, the dynamic mechanical analysis technique limits the in vitro characterization of soft tissues. However, a few in-vivo approaches can be employed to independently evaluate the tissue viscosity^54^. Additionally, higher frequency measurements were not performed in dynamic mechanical analysis (DMA) because of the inertial effects imposed by the rheometer utilized in the investigation. In a study done by^54^ a minimum frequency of 4 Hz and less than 50 Hz as the maximum was used for the DMA. As a result, it is challenging to compare the two approaches in various samples at high frequencies.

TWE is one of the essential modern dynamic elastography alternatives with significant advantages. Because this technique is focused on the propagation of shear waves through tissues, it is applicable not only for the in-depth organs but also with its radial propagation ability for applications on skin tumors. Axis-symmetric waves allow for precise examination of the mechanical functionality of soft tissue in cylindrical geometries, which is currently challenged by approaches to elastography in small organs. The TWE technique shown in this study is capable of transmitting and receiving shear waves at frequencies ranging from 100 Hz to 3 kHz. When compared to commercial elastography devices, this frequency range is advantageous. The Kelvin-Voigt model and its fractional derivative version successfully fit the cervical rheometric and TWE data from low to highest frequencies^10^. Validation is a critical step in any research project involving the computational modeling of physical phenomena. Many techniques, including experimental study, can be utilized to validate computational models. The initial stage was to prepare the phantoms, and after reviewing the relevant literature, it was determined that gelatin-agar was the best material to represent human tissue^55–58^. Different groups of phantoms representing normal and cancerous tissue were created to capture the range of viscosity parameters. Two different thicknesses of the first layer reflect the early stages of skin cancer^25^. Even viscoelastic parameters vary across individuals. The TWE probe was used to test the phantoms to measure the signal at the reception point and obtain velocity measurements by time-of-flight approach^9,10,21^. The experimental signals generated by the Ultrasonic Lab team’s TWE sensor demonstrated that the amplitude of signals decreases with depth, which is compatible with the scientific literature^59^. To validate the experimental work, Ultrafast Imaging carried out by the Verasonics Research System was set up^60–62^. The torsional wave velocity in the phantoms was measured using Ultrafast Imaging. After discovering that there is a good correlation between the TWE sensor and the method used in the literature, with a Pearson correlation coefficient (r) of 0.92 between the two approaches, The student’s T-test, and Mann-Whitney U-test (both with a 0.05 > p-value) results are 0.8188 and 0.5737, respectively. Evidence suggests that the two approaches are not significantly different from one another. For example, using TWE, the average velocity of Phantom 1 was 2.434 ± 0.121 m/s while utilizing the Ultrafast Imaging, the average velocity was 2.641±0.012 m/s. Following data analysis, determining the wave speed for each technique as a function of frequency and fitting curves with Eqs. (1) and (2). The mean and standard deviation of SP characteristics were calculated using three independent measurements. The SP parameters exhibit good agreement and correlation in both methods, as evidenced by the velocity measurements. A study of how various materials affect torsional wave velocity yields the following findings: when comparing phantoms 1, 3, and 5, the torsional wave velocity normally increases as the gelatin content increases. An increase in torsional wave velocity correlates with an increase in gelatin percentage in certain conditions^63,64^. Except for Phantom 11 and 12, where the oil percentages are 10% and 15%, most Phantoms maintain a consistent oil proportion of 5%. The torsional wave velocity between Phantom 10, 11, and 12 is slightly different; it increased from 10 to 11 and stayed constant between 11 and 12^64,65^. The velocity slightly increases as the percentage of agar rises. Comparing Phantom 1, 2, 3, 4, 5, 6 and 7 to Phantom 8, 9 and 10, we can see that the torsion velocity often increases when the agar percentage goes from 1 to 2%^66^. The reconstruction of the viscoelastic parameters has been established using the TWE methodology and the PIP method. Five parameters ($\alpha$1, $\eta$1, tick, $\alpha$2, and $\eta$2) were found for each phantom by fitting the experimental signals of bi-layer phantoms with the simulated signals of the SP-FDTD model. The signals are analyzed and compared using Pearson correlation, dynamic time warping (DTW), and time-frequency representation. Phantom 1, for instance, has a normalized DTW distance of 0.13 and a Pearson correlation coefficient of 0.99, which indicates that the two signals are reasonably well-matched^67–70^. According to an examination of the PIP data, gelatin percentage and $\eta$1 in the first layer have a positive relationship, as increasing gelatin percentage causes an increase in $\eta$1 value^39,64^. The $\alpha$1 parameter rises to its maximum value of 0.875 when the agar concentration in the first layer goes up from 1% to 2% (Phantoms 8, 9, and 10), and the $\eta$1 parameter also goes up^39,66^. In all phantom setups, the second layer’s thickness is constant. As a result, we cannot determine how it affects the parameters. Different phantom setups varied slightly in the $\eta$2 parameters of the second layer. As the percentage of gelatin in the first layer increases, the second layer’s $\eta$2 parameter also appears to rise. The $\alpha$1 parameter reduces to 0.85 in the first layer and 0.8 in the second layer when the oil percentage rises from 5 to 10% in the first layer (phantom 11), and the $\eta$1 parameter increases in the first layer and stayed constant when the oil percentage rises from 10 to 15% in the first layer (phantom 12)^39,64,66,71^. Finally, the correlation between the theoretical technique using Eqs. (1) and (2) for the first layer and the inverse problem method for reconstructing the parameters is found in order to validate the outcomes of the PIP method. Where the Pearson correlations for the two approaches $\eta$ and $\alpha$ are 0.83 and 0.93, respectively. This demonstrates good agreement and the capability to reconstruct the properties of skin tissue using the PIP method.

As a result, using the SP model with TWE is an effective method for identifying normal and cancerous tissue in the skin. Furthermore, it can diagnose any changes in the early stages. One key limitation of the TWE technique arises from its sensitivity to experimental conditions. Factors such as temperature variations and probe alignment can influence wave propagation, affecting the accuracy of our measurements. Precise control over these conditions is essential for reliable data acquisition. Additionally, the assumption of material homogeneity and isotropy in our model simplifies the analysis but does not fully capture the complexity of biological tissues, which are inherently heterogeneous and anisotropic. This simplification may limit the direct applicability of our findings to in vivo tissue characterization, necessitating further research to refine the TWE method for more accurate representation of biological tissue properties. The effects of inadequate cancer care are a reduced chance of survival, increased patient morbidity, and higher medical costs that contribute to preventable death and cancer disabilities. Early diagnosis improves cancer outcomes by offering treatment as early as possible and is thus an effective tool for public health in all settings^72^. TheSP-FDTD and torsional waves make skin cancer more easily identified at an early stage, resulting in a high recovery rate and cost savings. Also, in various applications, accurate modeling of viscoelastic tissue is critical. Cost and ease of use are also critical factors to consider. The SP model provides an alternative mathematical representation that concisely describes the reported viscoelastic properties of skin tissue and multilayered tissue such as cartilage or arterial wall. Understanding the changes in the mechanical properties of skin tissue can be used to detect most tumors in small organs, such as the mouth and glands. Torsional Waves Elastography is a modern, safe, and easy-to-use tool that opens a wider door to new applications benefiting people in many areas.

## Conclusion

In summary, the efficacy of the SP model combined with TWE for the characterization of skin cancer tissues was demonstrated in this study. The validation process was critical, beginning with the preparation of gelatin-agar phantoms that emulate the biomechanical properties of tissues. The TWE probe was used to acquire precise velocity data through the TOF method, and the Ultrafast Imaging setup confirmed the experimental results. Statistical analysis with the Mann-Whitney U-test and the Student’s T-test produced p-values of 0.8188 and 0.5737, respectively, indicating no significant difference between the TWE and PIP methods. The reconstruction of the viscoelastic parameters was successful, with five parameters ($\alpha _1$, $\eta _1$, thickness, $\alpha _2$, and $\eta _2$) identified for each phantom by fitting the experimental signals to the FDTD simulated SP model. The promising results of this study pave the way for the development of innovative diagnostic technologies for the early and precise detection of malignant tumors, with significant potential to improve patient outcomes. Looking ahead, we aim to refine the TWE technique to enhance its sensitivity and specificity in detecting various stages of tumor development. Future work will also explore the application of the SP model to other tissue types and investigate the integration of artificial intelligence to automate the analysis, enhancing the efficiency and accuracy of diagnoses. Moreover, building on the foundational studies conducted with phantom models, we plan to extend our research to include in vivo experiments. These studies will be crucial for validating the clinical applicability of our ultrasound elastography method in detecting conditions such as cancer and atherosclerosis in a real-world setting. By conducting in vivo studies, we aim to gather empirical data that will not only support the efficacy of our approach but also help in fine-tuning the technology for practical, clinical use. This progression towards in vivo applications is anticipated to significantly contribute to the field of medical imaging and diagnostics, offering new avenues for early and accurate detection of a wide range of pathologies.

### Patent information

The TWE device used in this study is protected by a patent application filed with the OEPM, with priority number P202130760.

## Data Availability

The datasets used and/or analysed during the current study available from the corresponding author on reasonable request.

## Code availability

The custom code and algorithms used in this study are available from the corresponding author upon reasonable request.
