# Author Correction: Using torsional wave elastography to evaluate spring pot parameters in skin tumor mimicking phantoms

**DOI:** 10.1038/s41598-024-69708-6

**Published:** 2024-08-14

**Authors:** Yousef Almashakbeh, Hirad Shamimi, Antonio Callejas, Guillermo Rus

**Affiliations:** 1 Department of Allied Engineering Sciences, Facility of Engineering, The Hashemite University, Zarqa, 13133 Jordan; 2 Department of Structural Mechanics, University of Granada, Granada, 18071 Spain; 3 Instituto de Investigación Biosanitaria, ibs.GRANADA, Granada, 18012 Spain; 4 Excellence Research Unit, “Modelling Nature” (MNat), Granada, 18071 Spain

Correction to: *Scientific Reports* 10.1038/s41598-024-66621-w, published online 11 July 2024

In the original version of this Article, Affiliation 1 was incorrect.

“Department of Allied Engineering Sciences, The Hashemite University, Zarqa 13133, Jordan.”

now reads

“Department of Allied Engineering Sciences, Facility of Engineering, The Hashemite University, Zarqa 13133, Jordan.”

In addition, the Acknowledgements section was incorrect.

 “Under grant 101096884, Listen2Future is co-funded by the European Union. Views and opinions expressed are however those of the author(s) only and do not necessarily reflect those of the European Union or Chips Joint Undertaking. Neither the European Union nor the granting authority can be held responsible for them. The project is supported by the CHIPS JU and its members (including top-up funding by Austria, Belgium, Czec Republic, Germany, Netherlands, Norway and Spain.”

now reads,

“Listen2Future is co-funded by the European Union under grant 101096884. Views and opinions expressed are however those of the author(s) only and do not necessarily reflect those of the European Union or Chips Joint Undertaking. Neither the European Union nor the granting authority can be held responsible for them. The project is supported by the CHIPS JU and its members (including top-up funding by Austria, Belgium, Czech Republic, Germany, Netherlands, Norway and Spain.”

The original Article has been corrected.

