# Author Correction: Using torsional wave elastography to evaluate spring pot parameters in skin tumor mimicking phantoms

**DOI:** 10.1038/s41598-024-76809-9

**Published:** 2024-10-24

**Authors:** Yousef Almashakbeh, Hirad Shamimi, Antonio Callejas, Guillermo Rus

**Affiliations:** 1 Department of Allied Engineering Sciences, Facility of Engineering, The Hashemite University, Zarqa, 13133 Jordan; 2 Department of Structural Mechanics, University of Granada, Granada, 18071 Spain; 3 Instituto de Investigación Biosanitaria, ibs.GRANADA, Granada, 18012 Spain; 4 Excellence Research Unit, “Modelling Nature” (MNat), Granada, 18071 Spain

Correction to: *Scientific Reports *10.1038/s41598-024-66621-w, published online 11 July 2024

In the original version of this Article, the Funding section was incomplete.

“This research was funded by Ministerio de Ciencia e Innovación grant numbers PID2020-115372RB-I00, PYC20 RE 072 UGR; Junta de Andalucía - Consejería de Universidad, Investigación e Innovación - proyecto P21.00182; Listen2Future funding by 101096884 in HORIZON-KDT-JU-2021-2-RIA and by PCI2022-135048-2 by Ministerio de Ciencia e Innovación; Proyectos Intramurales IBS.Granada INTRAIBS-2022-05. Ministerio de Universidades Ayudas para la recualifcación del sistema universitario español, MS2022-96 (Co funded by European Union Next GenerationEU/PRTR).”

now reads

“This research was funded by Grant PCI2022-135048-2 funded by MICIU/AEI/10.13039/501100011033 and by the European Union Next GenerationEU/PRTR, Ministerio de Ciencia e Innovación grant numbers PID2020-115372RB-I00, PYC20 RE 072 UGR; Junta de Andalucía - Consejería de Universidad, Investigación e Innovación - proyecto P21.00182; Listen2Future funding by 101096884 in HORIZON-KDT-JU-2021-2-RIA and by PCI2022-135048-2 by Ministerio de Ciencia e Innovación; Proyectos Intramurales IBS.Granada INTRAIBS-2022-05. Ministerio de Universidades Ayudas para la recualifcación del sistema universitario español, MS2022-96 (Co funded by European Union Next GenerationEU/PRTR).”

The original Article has been corrected.

